# Reduction of Silent Information Regulator 1 Activates Interleukin-33/ST2 Signaling and Contributes to Neuropathic Pain Induced by Spared Nerve Injury in Rats

**DOI:** 10.3389/fnmol.2020.00017

**Published:** 2020-02-12

**Authors:** Yanyan Zeng, Yu Shi, Hongrui Zhan, Wei Liu, Guiyuan Cai, Haili Zhong, Yaping Wang, Shangjie Chen, Shimin Huang, Wen Wu

**Affiliations:** ^1^Department of Rehabilitation, Zhujiang Hospital, Southern Medical University, Guangzhou, China; ^2^Department of Rehabilitation, The Fifth Affiliated Hospital of Sun Yat-sen University, Zhuhai, China; ^3^Department of Rehabilitation, Baoan Hospital, Southern Medical University, Shenzhen, China

**Keywords:** neuropathic pain, IL-33, ST2, SIRT1, inflammation

## Abstract

Emerging studies have demonstrated that interleukin (IL)-33 and its receptor ST2 act as key factors in inflammatory diseases. Moreover, accumulating evidence has suggested that cytokines, including tumor necrosis factor (TNF)-α and IL-1β, trigger an inflammatory cascade. SIRT1 has been shown to suppress the expression of inflammatory cytokines. However, the effects of SIRT1 on IL-33/ST2 signaling and initiation of the inflammatory cascade *via* modulation of TNF-α and IL-1β by IL-33 remain unclear. In the present study, we found that the dorsal root ganglion (DRG) IL-33 and ST2 were upregulated in a rat model of spared nerve injury (SNI) and intrathecal injection of either IL-33 or ST2 antibodies alleviated mechanical allodynia and downregulated TNF-α and IL-1β induced by SNI. In addition, activation of SIRT1 decreased enhanced DRG IL-33/ST2 signaling in SNI rats. Artificial inactivation of SIRT1 *via* intrathecal injection of an SIRT1 antagonist could induce mechanical allodynia and upregulate IL-33 and ST2. These results demonstrated that reduction in SIRT1 could induce upregulation of DRG IL-33 and ST2 and contribute to mechanical allodynia induced by SNI in rats.

## Introduction

Chronic pain, often characterized by allodynia, hyperalgesia, and spontaneous pain affects approximately one-third of the world’s population (Alford et al., 2008). In the United States, direct and indirect costs of chronic pain have been estimated to be approximately $100 billion annually, which is more than the combined costs of cancer, heart disease, and diabetes (Pizzo and Clark, 2012). It is well established that peripheral and central sensitizations are the basic mechanisms of chronic pain (Meacham et al., 2017). Inflammation has been extensively reported to be associated with peripheral and central sensitization (Ji et al., 2018). However, the type of inflammatory cytokine that triggers the inflammation cascade remains controversial.

Interleukin (IL)-33 belongs to a member of the IL-1 family and exerts its effects *via* binding to its receptor ST2 (Xu et al., 2019). It has been demonstrated that IL-33 plays a vital role in many inflammatory conditions, including septic shock (Ding et al., 2019), atherosclerosis (Buckley et al., 2019) and rheumatoid arthritis (Pinto et al., 2019). Furthermore, other studies have suggested that IL-33 modulates cutaneous hyper-nociception in inflammatory pain in mice (Verri et al., 2008) and has been implicated in activating astrocytes in the spinal cord in mouse models of bone pain (Zhao et al., 2013). However, the role of IL-33 in the dorsal root ganglion (DRG) in neuropathic pain remains unclear.

The silent information regulator 1 (SIRT1) is an NAD^+^-dependent deacetylase belonging to the SIRT family (Hattori and Ihara, 2016). Among SIRT family, SIRT1 has been validated to be most related in this family and function as deacetylating and regulating histones (Ling et al., 2018) as well as a wide range of non-histone substrates, such as NF-κB (Kauppinen et al., 2013), p53 (Nakamura et al., 2017), FOXO (Brunet et al., 2004), ERK (Han et al., 2017), peroxisome proliferator-activated receptor γ (PPARγ) and others (Kauppinen et al., 2013). With regulating this protein, SIRT1 plays a central role in regulating cellular processes, including apoptosis (Ling et al., 2017), cellular proliferation (Jablonska et al., 2016), and inflammation (Zhang et al., 2017). In the nervous system, SIRT1 suppress the neurodegenerative diseases such as Alzheimer’s disease and Parkinson’s disease *via* anti-apoptosis, anti-inflammation (Singh et al., 2017; Gomes et al., 2018). Recently, several studies have reported that the activation of SIRT1 in the spinal cord alleviates neuropathic pain induced by chronic constriction injury (CCI) surgery in rats and mice *via* inhibition of the inflammatory cascade (Shao et al., 2014; Lv et al., 2015). In addition, SIRT1 in the spinal cord epigenetically upregulates inflammasome NALP1 expression and contributes to the chronic pain induced by the chemotherapeutic drug bortezomib (Chen et al., 2018). However, whether SIRT1 modulates the inflammatory cytokine IL-33 remains unclear.

In the present study, we performed spared nerve injury (SNI) surgery in rats to establish a neuropathic pain model and hypothesized that downregulation of SIRT1 in the DRG induced by SNI enhances IL-33/ST2 signaling and triggers a downstream inflammatory cascade leading to mechanical allodynia.

## Materials and Methods

### Animals

Male Sprague-Dawley rats, weighing 200–250 g, were obtained from the Institute of Experimental Animals of Southern Medical University (Guangzhou, China; Approval number: SCXK 2016-0041). The animals were housed in standard cages in a temperature-controlled (24 ± 1°C) colony room under a 12 h light/dark cycle regimen, with *ad libitum* access to food and water. The experimental protocols were approved by the Southern Medical University Animal Care and Use Committee and were performed in accordance with the National Institutes of Health Guide for the Care and Use of Laboratory Animals.

### Surgery and Drug Administration

SNI model rats were developed in accordance with previously described procedures (Decosterd and Woolf, 2000). Briefly, after making an incision on the skin at the lateral surface of the thigh, a section was made directly through the biceps femoris muscle to expose the sciatic nerve and its three terminal branches, the sural, common peroneal, and tibial nerves. The SNI procedure involves axotomy and ligation of the tibial and common peroneal nerves but leaves the sural nerve intact. The common peroneal and tibial nerves were tightly ligated using 5.0 silk and transected distal to the ligation, removing approximately 4 mm of the distal nerve stump. Care was taken to avoid any damage to the nearby sural nerve. After surgery, all wounds were irrigated with sterile saline and closed in layers. In the sham group, an identical procedure was performed to expose the sciatic nerve and its three terminal branches, but without any nerve injury. For intrathecal delivery of the SIRT1 agonist SRT1720, the animals were implanted with catheters during the same surgery, as previously reported (Hirai et al., 2014). Briefly, a sterile catheter filled with saline was inserted through the lumbar (L) 5/6 intervertebral space, and the tip of the tube was positioned at the lumbosacral spinal level. Animals that exhibited hind limb paralysis or paresis after surgery were excluded. For animals without movement disorders, lidocaine (2%) was administered through the catheter to verify the intraspinal location. An immediate bilateral hind limb paralysis (within 15 s) lasting 20–30 min confirmed the correct catheterization. Animals without the aforementioned features were not used in the experiments that followed. The SIRT1 agonist SRT1720 and antagonist EX-527 were dissolved in DMSO and intrathecal administration at concentration of 15 mg/kg and 10 mg/kg respectively. IL-33 (rIL-33; 3626-ML) and ST2- neutralizing antibody (AF1004) were purchased from R&D Systems (Minneapolis, MN, USA). The rIL-33 and ST2 antibodies were diluted in sterile phosphate buffer solution (PBS).

### Behavioral Test

Mechanical sensitivity was assessed using von Frey hairs and the up-down method, as previously described (Chaplan et al., 1994). Briefly, after acclimatization to the testing environment for 2 h per day on three consecutive days, the rats were placed in separate transparent testing chambers positioned on a wire mesh floor. After a 10-min adaptation period, each stimulus consisted of a 2–3 s application of von Frey hairs to the middle of the plantar surface of the hind paws and the lateral surface of ipsilateral hind paws for SNI rats, with a 5-min interval between consecutive tests. Quick withdrawal or licking of the paw in response to the stimulus was considered a positive response. The operator performing the behavioral tests was blinded to the study design.

### Immunohistochemistry

The animals were deeply anesthetized using 50 mg/kg sodium pentobarbital (intraperitoneal) and perfused through the ascending aorta with saline, followed by 4% paraformaldehyde in 0.1 M phosphate buffer (4°C, pH 7.4), as previously described. After perfusion, the L4, L5, and L6 DRGs were removed and post-fixed in the same fixative for 3 h, which was subsequently replaced with 30% sucrose (in 0.1 M PBS) overnight. Frozen tissues were sectioned in the longitudinal plane with a thickness of 16 mm using a microtome and processed for immunofluorescence staining. All sections were blocked with 3% donkey serum in 0.3% Triton X-100 for 1 h at room temperature and incubated over two nights at 4°C with primary antibodies. After incubation with primary antibodies, the tissue sections were washed three times in 0.01 M PBS and then incubated in Cy3-conjugated donkey anti-rabbit IgG (diluted 1:300; Jackson ImmunoResearch, West Grove, PA, USA) for 1 h at room temperature. For double immunofluorescence staining, tissue sections were incubated with a mixture of anti-SIRT1 [1:200, Cell Signaling Technologies (CST), Danvers, MA, USA] antibody with neurofilament-200 [NF-200 (a marker for myelinated A-fibers), 1:200; Chemicon/Thermo-Fisher Scientific, Waltham, MA, USA], IB4 [FITC-conjugated (a marker for nonpeptidergic C-type neurons), 20 mg/ml (Sigma)], anti-calcitonin gene-related peptide [CGRP (a marker of peptidergic C-type neurons), 1:500, Abcam, Cambridge, MA, USA], IL-33 (1:300, Abcam, Cambridge, MA, USA), ST2 (1:400, Abcam, Cambridge, MA, USA) over two nights at 4°C. Except for isolectin-B4 (IB4)-treated tissue sections, all of the aforementioned sections were treated with a mixture of FITC and Cy3-conjugated secondary antibodies for 1 h at room temperature. The sections were rinsed with 0.01 M PBS three times and mounted on gelatin-coated slides and air-dried. The stained sections were examined using a fluorescence microscope (Leica, Wetzlar, Germany) and images were captured using a charge-coupled device spot camera.

### Western Blotting

Western blotting was performed according to the method described in a previous study (Hnasko and Hnasko, 2015). Briefly, L4–L6 DRG tissues of animals were removed and homogenized in 15 mmol/L Tris containing a cocktail of proteinase inhibitors after the animals were anesthetized with 50 mg/kg sodium pentobarbital (intraperitoneal). Next, the L4–L6 DRG lysates were prepared and separated using sodium dodecyl polyacrylamide gel electrophoresis and transferred to a polyvinylidene fluoride membrane. The membranes were then pre-incubated with blocking buffer for 1 h at room temperature. After incubating with diluted primary antibodies against SIRT1 (1:1,000, CST), IL-33 (1:1,000, Abcam), ST2 (1:1,000, Abcam), tumor necrosis factor (TNF)-α (1:1,000, Abcam), and/or β-actin (1:2,000, Abcam) overnight at 4°C, the membranes were incubated in horseradish peroxidase-conjugated secondary antibody for 1 h at room temperature. Finally, protein bands on the membranes were visualized using a commercially available enhanced chemiluminescence assay (Pierce, USA) according to the manufacturer’s instructions. The bands were subsequently quantified using a computer-assisted imaging analysis system.

### Statistical Analysis

All data are expressed as mean ± standard error of the mean (SEM). Statistical analysis was performed using SPSS version 20.0 (IBM Corporation, Armonk, NY, USA). For the behavior test, one- or two-way analysis of variance (ANOVA) with repeated measures, followed by a Tukey *post hoc* test, was performed. Western blot was analyzed using one-way ANOVA followed by the Turkey *post hoc* test. Differences with *p* 0.05 were considered statistically significant.

## Results

### IL-33 and Its Receptor ST2 in DRG Were Induced and Upregulated by SNI in Rats

**Figure 1:**
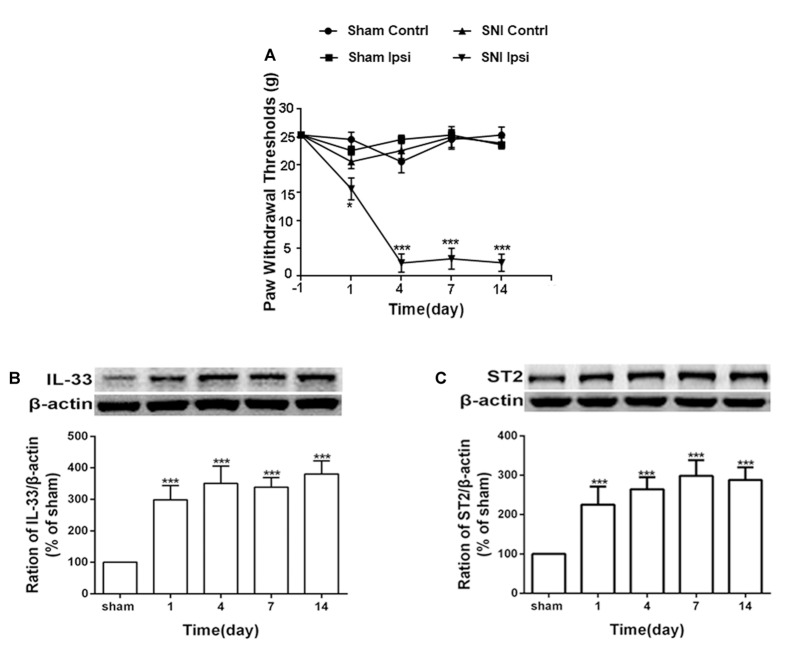
Spared nerve injury (SNI) induced mechanical allodynia and enhanced expression of interleukin (IL)-33 and ST2 in dorsal root ganglion (DRG) of rats. **(A)** SNI induced mechanical allodynia in the ipsilateral but not contralateral hind paw (*n* = 6), but the sham operation did not induce changes in bilateral hind paw(*n* = 6). **(B,C)** Compared with the sham rats, expression of IL-33 and its receptor ST2 increased in L4-L6 DRG since day 1 and persisted till day 14 after SNI (*n* = 4). *p < 0.05, ***p < 0.001 compared with the sham group.

Consistent with the results of a previous study (Boccella et al., 2018), the mechanical withdrawal threshold was significantly reduced in SNI rats (Figure 1A). To further study the role of IL-33/ST2 signaling in neuropathic pain induced by SNI, western blot assay was performed to analyze protein expression of IL-33 and ST2. As shown in Figures 1B,C, IL-33, and ST2 in DRG (L4-L6) was markedly increased on day 1 following SNI surgery and lasted at least until day 14.

### Enhanced IL-33 and ST2 Expression Contributed to Mechanical Allodynia Induced by SNI

**Figure 2:**
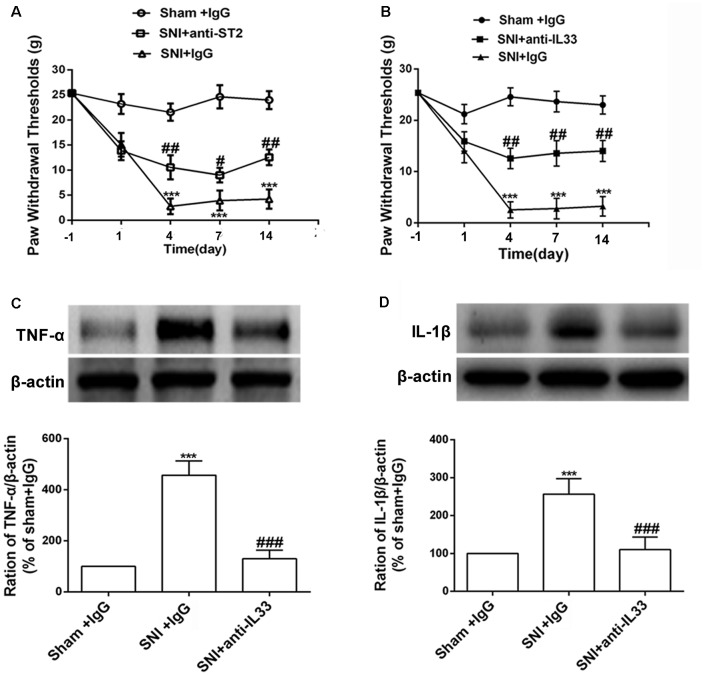
Intrathecal injection both IL-33 and its receptor ST2 neutralization antibody ameliorated mechanical allodynia and reversed the increased expression of tumor necrosis factor (TNF)-α and IL-1β. **(A,B)** Intrathecal application of IL-33 or ST2 neutralization antibody alleviated SNI-induced mechanical allodynia but IgG treatment did not show effects on mechanical allodynia induced by SNI. The dose of IL-33 or ST2 neutralization antibody was 100 ng for consecutive 10 days and 250 ng for consecutive 10 days, respectively. The time point for intrathecal injection was 15 min before SNI surgery, *n* = 6/group. **(C,D)** Upregulation of TNF-α and IL-1β was blocked after intrathecal injection of IL-33 neutralization antibody on day 7 (*n* = 4/group). ***p < 0.001 compared with sham group, ^#^*p* < 0.05, ^##^*p* < 0.01, ^###^*p* < 0.001 compared with SNI+IgG group.

We determined whether the increased expression of IL-33 and ST2 was involved in the development of mechanical allodynia in SNI rats. We investigated the behavior response after intrathecally injecting IL-33 and ST2 neutralizing antibodies. The results revealed that both IL-33 and ST2 neutralizing antibodies alleviated mechanical allodynia induced by SNI surgery for seven consecutive days (Figures 2A,B). Studies have reported that TNF-α and IL-1β activate the inflammatory cascade (Cavaillon et al., 2003; Iwawaki, 2017) and that IL-33 may also play an important role in it. To determine the role of IL-33 in the inflammation, we used the western blot assay to quantify the expression of TNF-α and IL-1β after intrathecal administration of IL-33 antibodies. The results revealed that both TNF-α and IL-1β levels were significantly decreased in the DRG (Figure 2).

### Reduction of SIRT1 Is Involved in the Mechanical Allodynia Induced by SNI

**Figure 3:**
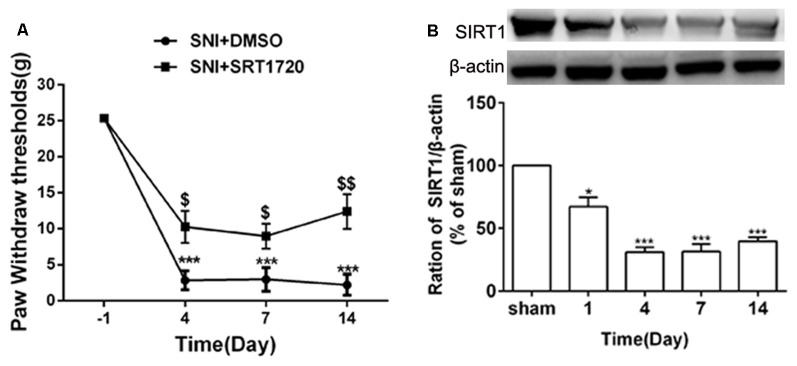
SNI reduced expression of DRG SIRT1 and intrathecal administration of SIRT1 agonist alleviated SNI-induced mechanical allodynia. **(A)** Intrathecal injection of SIRT1 agonist (15 mg/kg) 15 min before SNI surgery for consecutive 10 days alleviated the mechanical allodynia. **(B)** The time courses of the changes in the expression of SIRT1 after SNI surgery. *p < 0.05, ***p < 0.001 compared with sham group, ^$^*p* < 0.05, ^$$^*p* < 0.01 compared with SNI+DMSO group.

SIRT1 plays an important role in synaptic plasticity and chronic pain. The levels of SIRT1 protein in the DRG started to decrease on day 1 following SNI surgery, which lasted until at least day 14 (Figure 3B). Meanwhile, behavior testing demonstrated that intrathecal administration of the SIRT1 agonist SRT1720 remarkably decreased the paw withdraw thresholds induced by SNI in rats (Figure 3A).

### SIRT1 Is Expressed in DRG Neurons

**Figure 4:**
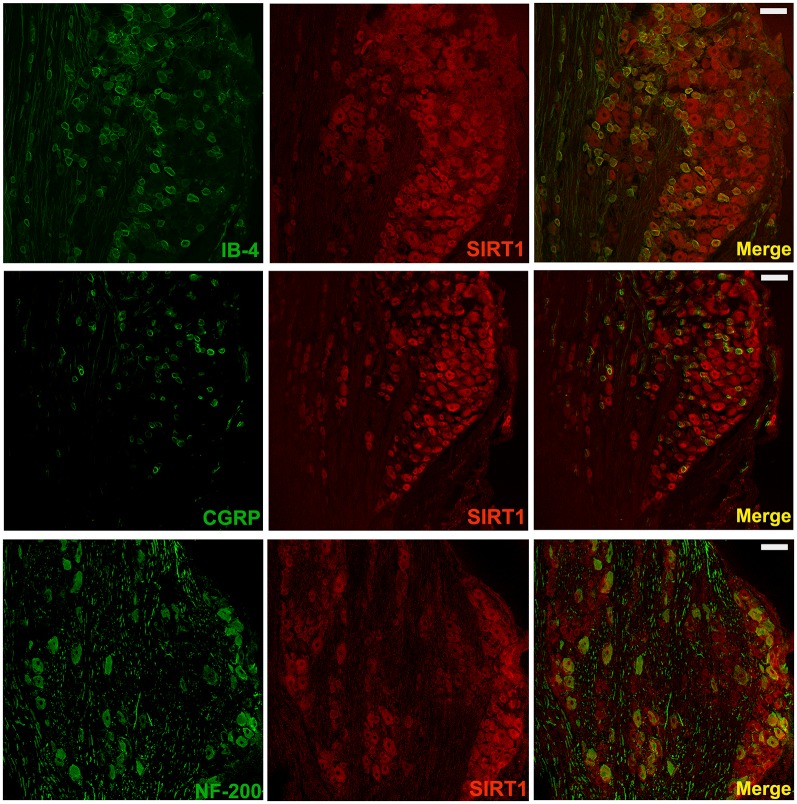
SIRT1 in DRG mainly expressed in neurons of rats. Double immunofluorescence staining showed that SIRT1 expressed in peptidergic neurons (CGRP marked) and non-peptidergic (IB-4 and NF-200 marked) neurons in DRG. Scale bar 200 μm.

It has been reported that SIRT1 is only expressed in neurons in the spinal cord; however, the distribution of SIRT1 in DRG remains unclear. To further verify the distribution of SIRT1 in the DRG of rats, double immunofluorescent staining was performed. As shown in Figure 4, SIRT1 was primarily expressed in NF200-positive cells (large-diameter neurons), IB4-positive cells, and CGRP-positive cells (small- and medium-diameter neurons).

### Activation of SIRT1 Downregulated IL-33/ ST2, TNF-α, and IL-1β

**Figure 5:**
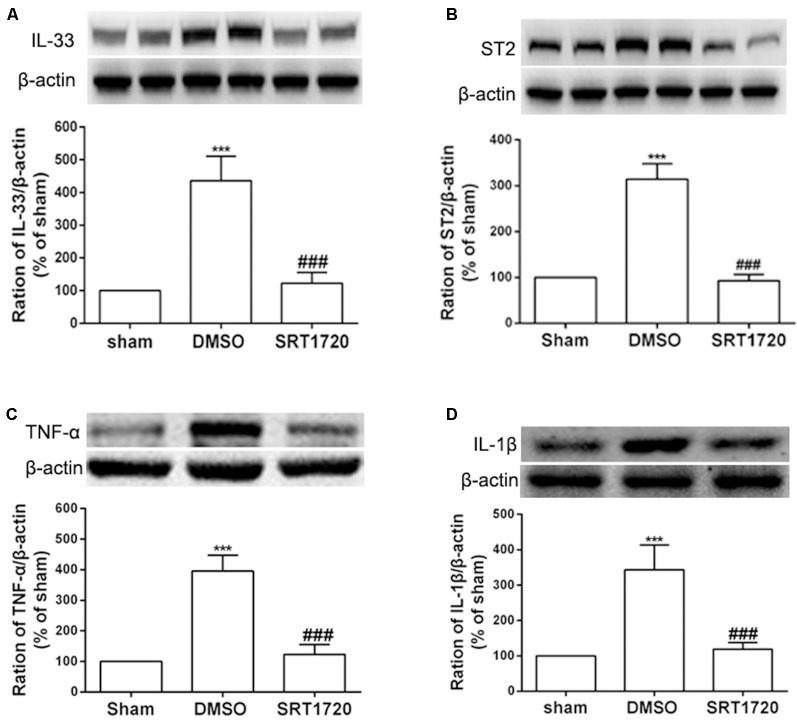
The increased expression of IL-33, ST2, TNF-α, and IL-1β were retarded by intrathecal injection of SIRT1 agonist SRT1720. ** (A,B)** Application of SRT1720(i.t.) reduced the expression of IL-33 and its receptor ST2 after SNI surgery on day 7. **(C,D)** Inflammatory cytokines TNF-α and IL-1β were significantly downregulated by delivery of SRT1720(i.t.) on day 7, *n* = 4/group, ***p < 0.001 compared with the sham group, ^###^*p* < 0.001 compared with SNI+DMSO group.

Several recent studies have presented evidence that SIRT1 mediates chronic pain through modulation of inflammation in the spinal cord. Thus, we determined whether SIRT1 regulated the DRG IL-33/ST2 signaling in rats with SNI-induced neuropathic pain. The double immunofluorescence staining showed that SIRT1 colocalized with IL-33 and ST2 (Supplementary Figure S1). As shown in Figures 5A,B, DRG IL-33 and its receptor ST2 were remarkably downregulated on day 7 following intrathecal injection of the SIRT1 agonist SRT1720. Similarly, the expression of the inflammatory cytokines TNF-α and IL-1β was reduced on day 7 after the injection of SRT1720 (Figures 5C,D). Furthermore, the enhanced acetylation of NF-κB was significantly alleviated by SRT1720 but have no effects on *p*-ERK (Supplementary Figure S2).

### SIRT1 Antagonist EX527 Induced Mechanical Allodynia in Naïve Rats

**Figure 6:**
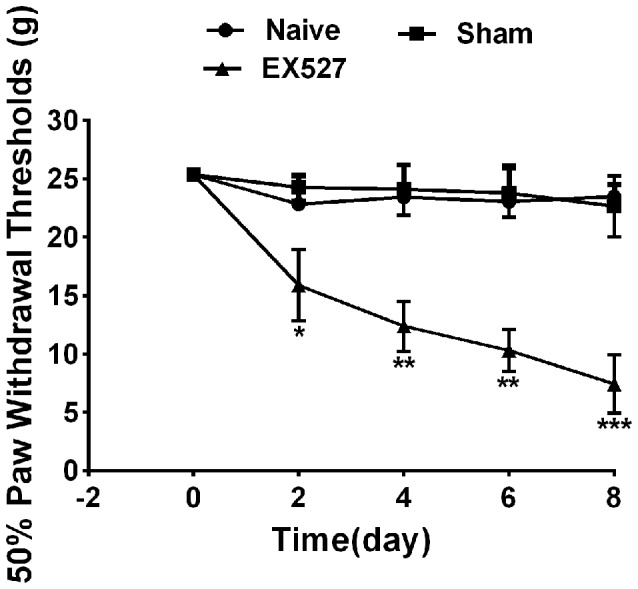
Continuous intrathecal injection of SIRT1 agonist EX527 at a dose of 10 mg/kg for successive 5 days induced mechanical allodynia in naïve rats. *n* = 6/group, *p < 0.05, **p < 0.01,***p < 0.001 compared with sham group.

To further define the effects of SIRT1 in DRG on pain behavior, the rats were intrathecally administered the SIRT1 antagonist EX-527 at a dose of 10 mg/kg for five consecutive days. A mechanical allodynia behavior test using von Frey filaments demonstrated that the paw withdrawal threshold was decreased in naïve rats from day 2 and lasted until day 8 after EX-527 treatment. However, the naïve rats that were administered saline exhibited no significant mechanical allodynia compared with naïve rats. Therefore, the EX-527 treatment in naïve rats induced mechanical allodynia (Figure 6).

### Inactivation of SIRT1 Can Enhance the Expression of IL-33 and ST2 in Naïve Rats

**Figure 7:**
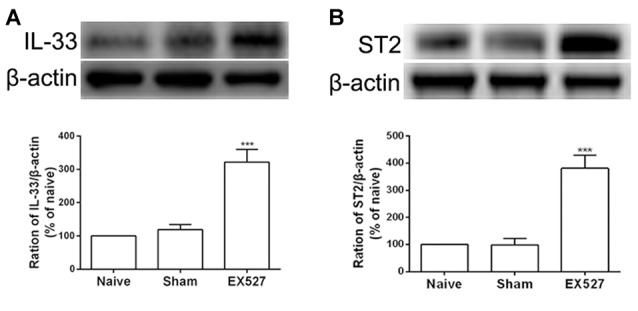
Continuous intrathecal injection of SIRT1 antagonist EX-527 upregulated DRG IL-33 and ST2. **(A,B)** The expression of IL-33 and ST2 significantly upregulated on day 5 after continuous delivery EX-527 in naïve rats. *n* = 4/group, ***p < 0.001 compared with the sham group.

Having observed that the SIRT1 antagonist contributed to mechanical allodynia in naïve rats, we tested whether the inactivation of SIRT1 modulated the expression of IL-33 and ST2. The results of the western blot analysis demonstrated that both IL-33 and ST2 proteins were upregulated on day 5 after intrathecal administration of EX-527 compared with saline treatment in naïve rats (Figures 7A,B).

## Discussion

In the present study, we found that the expression of IL-33 and its receptor ST2 in DRG increased after SNI surgery. Intrathecal administration of both IL-33 and ST2 antibodies alleviated mechanical allodynia induced by SNI. Intrathecal injection of IL-33 antibody and rat recombinant IL-33 decreased the enhanced expression of TNF-α and IL-β in SNI rats and induced the expression of TNF-α and IL-β in naïve rats, respectively. In addition, SNI surgery reduced the expression of SIRT1 in DRG neurons, and intrathecal injection of the SIRT1 agonist SRT1720 ameliorated mechanical allodynia and reversed the upregulation of IL-33 induced by SNI. Collectively, our results revealed a new mechanism in which reduction of SIRT1 activates IL-33/ST2 signaling and subsequently triggers the TNF-α and IL-1β inflammatory cascade, thus contributing to the mechanical allodynia induced by SNI.

### The Role of IL-33 in Triggering Inflammatory Cascade in Neuropathic Pain Following SNI

IL-33 is a cytokine in human endothelial cells that was discovered in 2003 (Baekkevold et al., 2003), it exerts its biological effects through the ST2/IL-1RAcP (IL-1 receptor accessory protein) receptor complex. Recent evidence has shown that IL-33 deficiency results in reduced innate papain-induced lung inflammation (Oboki et al., 2010). More recent studies have suggested that IL-33/ST2 contributes to the development of pain. For example, the expression of spinal IL-33 and ST2 was enhanced in mice with formalin-induced inflammatory pain (Zarpelon et al., 2013). Furthermore, activation of spinal IL-33 and ST2 has been reported to contribute to bone cancer pain (Zhao et al., 2013). In this study, we first found that the expression of DRG IL-33 and ST2 increased in a rat model of neuropathic pain induced by SNI. Intrathecal administration of IL-33 and ST2 antibodies alleviated mechanical allodynia. It has been firmly established that inflammatory cytokines are involved in the development and maintenance of neuropathic pain (Old et al., 2015; Ronchetti et al., 2017). Both TNF-α and IL-β have been reported to be cytokines that trigger the inflammatory cascade (Rider et al., 2011; Zelová and Hošek, 2013). In our study, we found that intrathecal administration of IL-33 and ST2 antibodies in SNI-treated rats could reduce the enhanced expression of TNF-α and IL-β. These results demonstrated that SNI could activate the IL-33/ST2 signaling pathway in DRG, subsequently trigger the inflammatory cascade, and contribute to the mechanism of allodynia.

### SIRT1 in DRG Contributes to the Activation of IL-33/ST2 Signaling Following SNI

Accumulating evidence has demonstrated that SIRT1 modulates the expression of inflammatory cytokines *via* targeting nuclear factor (NF)-κB (Yeung et al., 2004; Kauppinen et al., 2013). A recent study revealed that SRT1720 ameliorated chronic pain induced by chronic CCI through the regulation of spinal cord inflammation (Lv et al., 2015). Similarly, we found that intrathecal injection of the SIRT1 agonist SRT1720 suppressed the upregulation of TNF-α and IL-1β in the DRG and alleviated mechanical allodynia in SNI-treated rats. We also observed that the reduction of SIRT1 in DRG neurons contributed to the activation of the IL-33/ST2 signaling pathway. SIRT1 is an important deacetylase that directly deacetylates NF-κB (Deng et al., 2017). In addition, IL-33 can activate NF-κB through binding to ST2 (Numata et al., 2016). In our study, we observed that SRT1720 dramatically reduced the acetylation of NF-κB p65 induced by SNI. It is possible that the reduction of SIRT1 in the DRG increased acetylated NF-κB, upregulated IL-33, and triggered the inflammatory cascade, which may have played a vital role in the development of mechanical allodynia induced by SNI surgery.

Collectively, our results demonstrate that IL-33 in DRG and its receptor ST2 upregulated and modulated the expression of TNF-α and IL-1β in neuropathic pain induced by SNI. In addition, we observed that the reduction of DRG SIRT1 activated IL-33/ST2 signaling and contributed to mechanical allodynia in SNI rats. These results may suggest a new potential therapeutic target for neuropathic pain.

## Data Availability Statement

The raw data supporting the conclusions of this article will be made available by the authors, without undue reservation, to any qualified researcher.

## Ethics Statement

The animal study was reviewed and approved by Southern medical university animal protection and use committee.

## Author Contributions

YZ conceived and designed the experiments. YZ, YS, HZha, WL, GC, HZho, YW, and SH performed the experiments. YZ and YS collected and analyzed the data and wrote the article. HZha and WL contributed the reagents, materials and analysis tools. WW and SC provided the financial support. The first communication author in this article is WW, and the second communication author is SC.

## Conflict of Interest

The authors declare that the research was conducted in the absence of any commercial or financial relationships that could be construed as a potential conflict of interest.

## Supplementary Material

FIGURE S1 The colocalization of SIRT1/IL-33 and SIRT1/ST2. Double immunofluorescence staining showed that SIRT1 colocalized with IL-33 **(A–C)** and ST2 in DRG **(D–F)**. Scale bar 100 μm.

FIGURE S2 SIRT1 agonist reduced the acetylation of NF-κB p65 but had no effects on p-ERK. **(A)** The enhanced acetylation of NF-κB p65 in DRG was significantly alleviated in SNI rats by intrathecal administration SIRT1 agonist SRT1720 (*n* = 4/group). **(B)** SIRT1 agonist SRT1720 showed no effects on the increased *p*-ERK in DRG of SNI rats (*n* = 4/group). ****p* < 0.001 compared with the sham group, ^##^*p* < 0.01 compared with the SNI+DMSO group.
