# Supplementary figures and images for: Reduction of Silent Information Regulator 1 Activates Interleukin-33/ST2 Signaling and Contributes to Neuropathic Pain Induced by Spared Nerve Injury in Rats
Source: Front Mol Neurosci. 2020 Feb 12;13:17. doi: 10.3389/fnmol.2020.00017 (PMC7028692; doi:10.3389/fnmol.2020.00017)

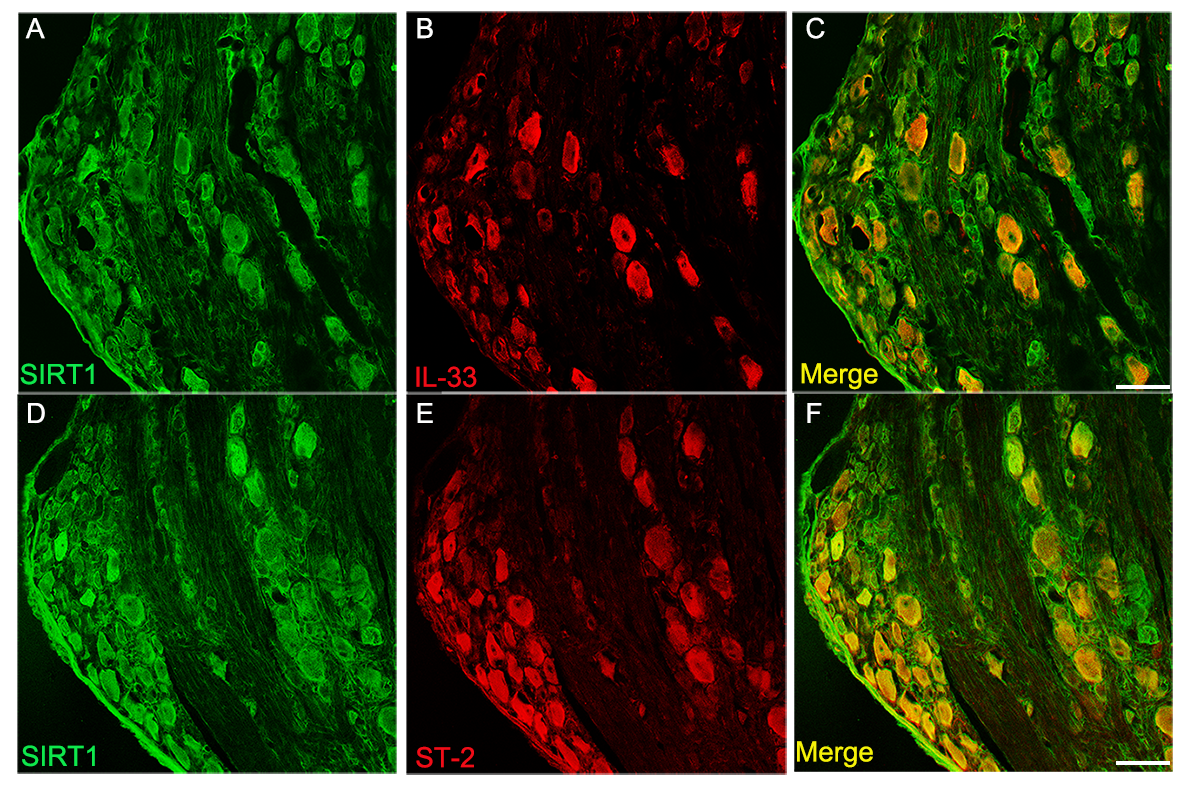

Supplement: FIGURE S1 — The colocalization of SIRT1/IL-33 and SIRT1/ST2. Double immunofluorescence staining showed that SIRT1 colocalized with IL-33 (A–C) and ST2 in DRG (D–F). Scale bar 100 μm. [file Image_1.tif]

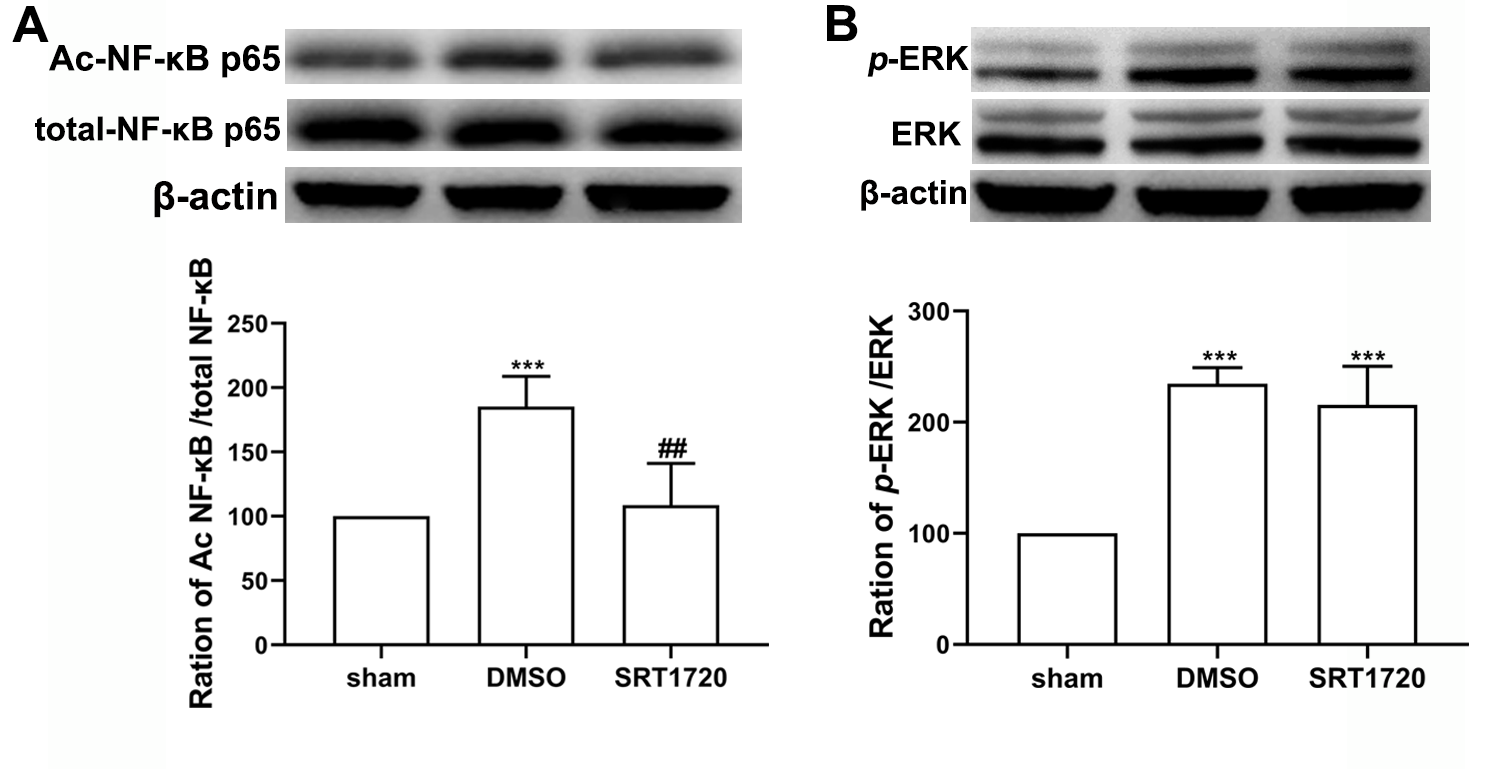

Supplement: FIGURE S2 — SIRT1 agonist reduced the acetylation of NF-κB p65 but had no effects on p-ERK. (A) The enhanced acetylation of NF-κB p65 in DRG was significantly alleviated in SNI rats by intrathecal administration SIRT1 agonist SRT1720 (n = 4/group). (B) SIRT1 agonist SRT1720 showed no effects on the increased p-ERK in DRG of SNI rats (n = 4/group). ***p < 0.001 compared with the sham group, ##p < 0.01 compared with the SNI+DMSO group. [file Image_2.tif]
